# Potential of advanced microporous zeolites and mesoporous materials derived from natural precursors as supports for iron phosphide catalysts in bio-jet fuel production from palm oil (*Elaeis guineensis*)†

**DOI:** 10.1039/d5ra02133b

**Published:** 2025-06-10

**Authors:** Worapak Tanwongwan, Ruttasart Sartsamai, Rungnapa Kaewmeesri, Kajornsak Faungnawakij, Nuwong Chollacoop, Suttichai Assabumrungrat, Masayoshi Fuji, Apiluck Eiad-ua

**Affiliations:** a School of Integrated Innovative Technology, King Mongkut's Institute of Technology Ladkrabang (KMITL) Ladkrabang Bangkok 10520 Thailand apiluck.ei@kmitl.ac.th +66897815665; b National Nanotechnology Center (NANOTEC), National Science and Technology Development Agency (NSTDA) Khlong Luang Pathum Thani 12120 Thailand; c National Energy Technology Center (ENTEC), National Science and Technology Development Agency (NSTDA) Khlong Luang Pathum Thani 12120 Thailand; d Department of Chemical Engineering, Faculty of Engineering, Chulalongkorn University Phatumwan Bangkok 10330 Thailand; e Advanced Ceramic Center, Nagoya Institute of Technology Tajimi Gifu 5007-0033 Japan

## Abstract

Iron phosphide (FeP) has emerged as an efficient catalyst for converting palm oil, a biomass-derived feedstock, into bio-jet fuel through the hydrocracking process. The catalytic performance of FeP is strongly influenced by the choice of support material. In this study, microporous MWW-type zeolites (MCM-22 and MCM-36) and mesoporous materials (MCM-41 and MCM-48) were successfully synthesized from entirely natural precursors, silica derived from rice husk and aluminosilicate gel extracted from kaolin clay, *via* a hydrothermal method, and employed as supports for FeP catalysts. Among these materials, MCM-22 zeolite exhibited the highest microporosity, followed by zeolite MCM-36, resulting in superior acidity compared to the mesoporous materials, MCM-41 and MCM-48. FeP supported on MCM-22 (FeP/MCM-22) demonstrated the best catalytic performance, liquid hydrocarbon yield (∼33%), and bio-jet selectivity (∼78%) were obtained, outperforming FeP/MCM-36, FeP/MCM-41, and FeP/MCM-48. This is due to its high surface area of micropores (∼187 m^2^ g^−1^) and the excellent acidity of this zeolite, which helped prevent FeP overloading and promote uniform metal distribution. Furthermore, it exhibited remarkable stability and reusability, with performance improving over three consecutive reaction cycles, LHCs yield increasing to 50% and bio-jet selectivity stabilizing at about 83%, attributed to enhanced acidity accessibility and progressive formation of the FeP active phase.

## Abstract 2

Synthesis of microporous zeolites (MCM-22 and MCM-36) and mesoporous materials (MCM-41 and MCM-48) from natural extracts for supporting FeP catalyst on the conversion of palm oil to bio-jet fuel.

## Graphical abstract

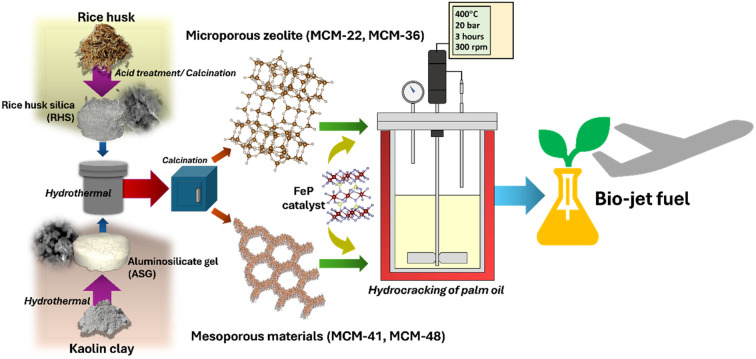

## Introduction

Air transportation is currently one of the most crucial sectors for global connectivity and economic development. According to the 79th IATA Annual General Meeting (AGM) held in Istanbul, Turkey, the sector's profit was estimated to reach USD 9.8 billion in 2023, with a net profit margin of 1.2%. The International Air Transport Association (IATA) further projected that airline operating profits could grow to USD 22.4 billion, with an annual revenue growth of 9.7%. Meanwhile, industry expenses are expected to rise by 8.1% annually, and cargo volume is estimated to reach 57.8 million tons.^1^ Among all operating costs, jet fuel remains a major expense in the aviation industry. According to IATA's Fuel Fact Sheet (mid-2023), the average cost of jet fuel increased by approximately 75% in 2022 to USD 136 per barrel, bringing the global fuel bill to USD 215 billion in 2023 – equivalent to roughly 28% of total operational costs.^2^ In parallel, growing concerns over greenhouse gas emissions have added urgency to the development of more sustainable aviation fuels. The large-scale consumption of jet fuel has been linked to significant carbon emissions, prompting global efforts toward eco-friendly alternatives. One promising strategy involves upgrading waste cooking oil or vegetable oils into bio-jet fuel *via* hydrocracking processes.^3^ Among various feedstocks, palm oil (*Elaeis guineensis*) has gained particular attention due to its high productivity and widespread cultivation in Southeast Asia, particularly in Indonesia, Malaysia, and Thailand. Metal phosphides have emerged as effective catalysts for hydrocracking, with iron phosphide (FeP) gaining interest due to its promising catalytic performance and relatively low cost.^4^ However, the inherently low surface area of bulk metal phosphides often limits their activity. This challenge can be addressed by dispersing the active phase onto support materials. Acidic supports, especially zeolites, not only enhance the surface area and porosity of the catalyst but also contribute catalytic activity through their Brønsted acid sites.^5^ Advanced mesoporous materials, such as hexagonally MCM-41 and cubic MCM-48, have been extensively investigated for such applications. For instance, in a study of Alsobaai *et al.*^6^ involving NiW/MCM-48 for the hydrocracking of gas oil, increased alumina content in MCM-48 was found to enhance catalyst activity due to higher acidity, although excessive alumina led to catalyst deactivation *via* coking. Similarly, Lu *et al.*^7^ demonstrated that the Si/Al ratio in Pt/Al-MCM-41 catalyst had a pronounced effect on its acidity, which in turn influenced the performance of bio-alkanes’ hydrocracking and selectivity of bio-jet fuel, highlighting the critical role of acidity in product distribution and fuel quality. In contrast, the application of microporous materials in the MCM (Mobil Composition of Matter) family, such as zeolites MCM-22 and MCM-36, has been relatively underexplored for hydrocracking purposes. Although Lallemand *et al.*^8^ reported that Ni/MCM-22 showed lower activity than Ni/MCM-36 in ethylene oligomerization due to its microporous structure and higher acid site density, these same characteristics may offer advantages in reactions requiring strong acidity, such as hydrocracking of large molecules. This suggests that the catalytic potential of microporous MCM-type zeolites warrants further investigation in the context of biofuel production. Moreover, the common synthesis of these zeolites usually requires commercial silica and alumina sources. Although some researchers have adapted rice husk silica as a silica precursor or used natural kaolin clay as an alumina source, the combination of both natural sources in a single synthesis has been rarely reported.^9^ Based on these motivations, this work aims to synthesize microporous zeolites (MCM-22 and MCM-36) and mesoporous materials (MCM-41 and MCM-48) using precursors derived entirely from natural extracts, and to evaluate their potential as support materials for FeP catalysts in the production of bio-jet fuel from palm oil.

## Experimental

### Materials and chemicals

Rice husk waste from agriculture was collected from Nonthaburi province while kaolin clay was obtained from Lampang province, both from Thailand. Sodium hydroxide pellet (NaOH), orthophosphoric acid 85%, hydrochloric acid 37%, and Fe(NO_3_)_3_·9H_2_O were purchased from Carlo Erba. Hexamethyleneimine (HMI), cetyltri-methylammonium chloride (CTMACl), and tetrapropylammonium hydroxide (TPAOH) were bought from Sigma Aldrich. Tetraethyl orthosilicate (TEOS), cetyltrimethylammonium bromide (CTAB), and tetramethylammonium hydroxide (TMAOH) were acquired from Acros Organics. Palm oil, as a reaction feedstock was purchased from a local market by choosing to use the Morakot brand oil. Finally, cyclohexane (85%) and ethanol (A. R. grade) were bought from RCI Labscan.

### Preparation of silica from rice husk and aluminosilicate gel from kaolin clay

Based on the methodology described in our previous study,^9^ rice husk was initially washed with deionized water to remove surface impurities and subsequently dried at 100 °C. The material was treated by stirring in 3 M HCl solution at 100 °C for 2 h. After acid treatment, the solid was filtered, thoroughly washed with deionized water until neutral, and dried overnight at 100 °C. Finally, the sample was calcined in a muffle furnace at 800 °C for 12 h to obtain white rice husk silica (RHS).

Meanwhile, kaolin clay was ground and sieved to obtain particles smaller than 90 μm. The material was then subjected to hydrothermal treatment at 200 °C for 8 h in 8 M sodium hydroxide solution to induce structural breakdown of the kaolinite framework. Upon completion, the solid phase was separated by filtration, thoroughly washed with deionized water until neutral, and dried at 100 °C for 12 h. The alkaline filtrate from the hydrothermal step was also collected. Subsequently, the dried solid was stirred in 3 M HCl solution, followed by filtration to recover the acid-soluble components. The acidic filtrate was then neutralized to pH 7 using the previously collected alkaline mother liquor. A white aluminosilicate gel (ASG) was formed upon neutralization and subsequently dried in an oven to obtain an aluminosilicate gel (ASG).

### Synthesis of MCM-22 and MCM-36 zeolite

Aluminosilicate gel (ASG), serving as the primary alumina precursor, was dissolved in NaOH solution under continuous stirring. Subsequently, rice husk silica (RHS) was introduced into the system, followed by the slow addition of hexamethyleneimine (HMI) as a structure-directing agent, which was added dropwise over 30 min. The molar composition of the synthesis gel was maintained at Na/Si = 0.2, HMI/Si = 0.35, and H_2_O/Si = 20, based on the literature,^10^ while the RHS (Si) to ASG ratio was fixed at 3 by weight. Hydrothermal crystallization was then conducted at 150 °C for 7 days under stirring. The resulting solid product, MCM-22(P), was recovered by filtration, thoroughly washed with deionized water, and dried. To achieve MCM-22 zeolite, the MCM-22(P) was calcined at 540 °C for 20 h

The MCM-22(P) obtained from the previous step was also used as a precursor for the synthesis of MCM-36 zeolite. The procedure was adapted from the method reported by Zhang *et al.*^11^ with slight modifications. The starting mixture for MCM-36 preparation consisted of MCM-22(P), deionized water, cetyltrimethylammonium chloride (CTMACl), and tetrapropylammonium hydroxide (TPAOH) in a weight ratio of 1 : 3 : 1 : 0.3. The mixture was stirred at 80 °C for 72 h to obtain a swollen form of MCM-22(P), which was then washed with 50 ml of deionized water and dried at room temperature. The swollen MCM-22(P) was subsequently mixed with tetraethyl orthosilicate (TEOS) at a weight ratio of 1 : 5 and stirred at 80 °C for 24 h. The resulting colloidal product was filtered, washed, and dried at 100 °C overnight. Finally, the structure-directing agents were removed by calcination at 540 °C for 6 h to obtain MCM-36 zeolite.

### Synthesis of mesoporous materials MCM-41 and MCM-48

Mesoporous MCM-41 and MCM-48 were also synthesized for the comparison study. RHS and aluminosilicate gel extracted from kaolin clay were also applied as silica and alumina sources while the procedure for MCM-41 was based on a study of Borade and Clearfield.^12^ Firstly, a solution (A) was prepared by dissolving RHS and tetramethylammonium hydroxide (TMAOH) in DI water and stirring at room temperature for 15 min. Simultaneously, a solution (B) was achieved by dissolving an ASG and cetyltrimethylammonium chloride (CTMACl) into NaOH solution and stirring for 15 min at ambient temperature. Then, solutions (A) and (B) were mixed and continuously stirred for 15 min. The weight ratio of all reagents is ASG : 3 RHS : 0.12 TMAOH : 0.25 Na : 1 CTMACl : 100H_2_O. A hydrothermal process was performed at 150 °C for 7 days. The product was washed with DI water, filtered, and dried at 100 °C overnight. Finally, a structure directing agent was eliminated by calcined at 550 °C for 10 h.

The preparation of MCM-48 was based on Kumar *et al.*^13^ with a few adaptations. CTAB, as a structure-directing agent, was dissolved in NaOH solution by stirring. After that, RHS and ASG were added in sequence. The mixture that contains ASG : 3 RHS : 0.6 Na : 11.85 CTAB : 100H_2_O, by weight was stirred at room temperature for 1 h and transferred into an autoclave reactor. Like all previous conditions for this study, the hydrothermal was performed at 150 °C for 7 days and a product was washed and dried at the same states. Finally, calcination in a muffle furnace eliminated the structure directing agent at 550 °C for 6 h.

### Catalyst preparation

Firstly, Fe(NO_3_)_3_·9H_2_O was stirring dissolved in DI water for 1 h. Then, the phosphoric acid solution slowly dropped into a system where the molar ratio of Fe and phosphorus was calculated to 1. Supports material was loaded into the solution where the ratio of metal was set at 10% wt. The mixture was continuously stirred in the ambient atmosphere for 3 h and moved to place in the oven at 100 °C overnight. Finally, the dried precursor was calcined at 800 °C for 5 h in a muffle furnace to achieve the catalyst.

### Characterization

The crystallography of all catalysts was observed by X-ray diffraction technique (SmartLab, Rigaku) and perceiving a functional group by Fourier-transform infrared spectroscopy (Spectrum Two, PerkinElmer). The surface and pore properties were analyzed by N_2_ adsorption–desorption analysis technique (autosorb iQ, Quantachrome instruments), all samples were degassed for 6 h at 300 °C with a heating rate of 10 °C min^−1^ before assessment. The thermal stability of materials was evaluated by a Thermogravimetric analyzer (TG 209 F3 Tarsus, NETZSCH) under the N_2_ atmosphere. The acidity and reduction behavior of all samples were studied by a chemisorption analyzer (ChemStar™ TPx, Quantachrome Instrument). A catalyst's particle distribution and internal structure were noticed through the transmission electron microscope (JEM-2100plus, JEOL). Lastly, scanning electron microscopy with energy-dispersive X-ray spectroscopy (EVO MA10, Zeiss) was applied to observe the morphology and composition of materials.

### Bio-jet fuel production

Prior to a reaction performed, all catalysts were treated *via* the H_2_-TPR technique at 600 °C with 50 ml min^−1^ of H_2_ gas. The reaction was performed in the high-pressure Parr reactor. Firstly, 3.7 ml of palm oil and 0.3 g of catalyst were loaded into an autoclave while 40 ml of cyclohexane was filled as solvent. Air was eliminated from a system by hydrogen purges for 5 min and a hydrogen gas was pressed into a system for 20 bars. The reaction was processed at 400 °C for 3 h with 300 rpm of stirring. A liquid product was analyzed by Gas chromatography-mass spectrometer (GCMS-QP 2010 Ultra, SHIMADZU) with DB-1HT column (Agilent).

These equations were applied for a calculation of catalytic behaviors:1
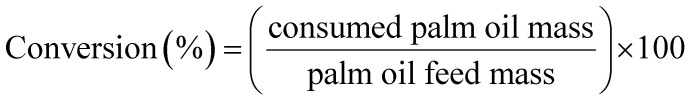
2
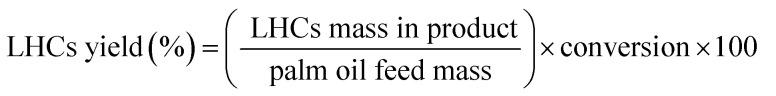
3
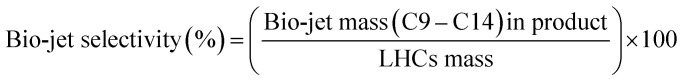


## Result and discussion

### Crystallization of microporous zeolites and mesoporous materials

**Fig. 1:**
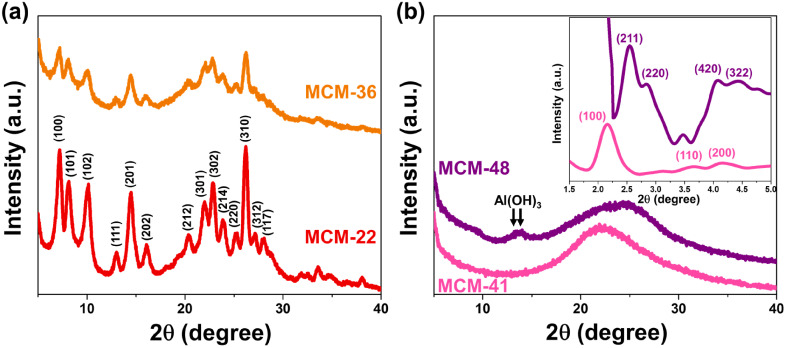
Crystallography of synthesized support materials (a) MWW-type zeolites and (b) mesoporous materials MCM-41 and MCM-48 (with the inset showing the small-angle XRD pattern).

The X-ray diffractogram of MWW-type zeolites (MCM-22 and MCM-36) confirms the structural formation of these zeolites,^14^ as shown in Fig. 1a. However, a difference in crystallinity was observed, with MCM-22 exhibiting the highest crystallinity at approximately 63%. The pillaring process significantly reduced the crystallinity of the MWW-type zeolite, with MCM-36 showing a lower crystallinity at around 29%. Notably, the intra-layer peak (100) of the MWW-type zeolite was significantly diminished in MCM-36 compared to MCM-22, indicating a decrease in the long-range order of the MWW structure.^15^ This reduction is attributed to the pillaring of the 10-membered ring (10 MR) crossing windows. Fig. 1b presents a wide-angle X-ray scattering curve, with the small-angle X-ray diffractogram shown in the subfigure. For MCM-41, small-angle reflection peaks (100), (110), and (200) confirm the formation of the MCM-41 structure.^16^ The observed weakening of the (100) and (200) planes suggests the significant presence of alumina in the MCM-41 structure, likely due to the agglomeration of Al^3+^ ions within the MCM-41 framework, which causes an expansion of its crystalline lattice.^17^ The wide-angle diffractogram displays a broad diffraction band in the range of 13–38°, consistent with the mesoporous silica framework (card no. 00-049-1712).^18^ The formation of MCM-48 is confirmed by the appearance of major peaks (211), (220), (420), and (322), which correspond to the *Ia*3*d* space group of MCM-48's cubic mesophases.^19^ However, in the wide-angle XRD, peaks of Al(OH)_3_ appear at the (−101) and (101) planes, indicating incomplete alumina consumption during the structure formation of this material.

### Chemical structure of microporous zeolites and mesoporous materials

**Fig. 2:**
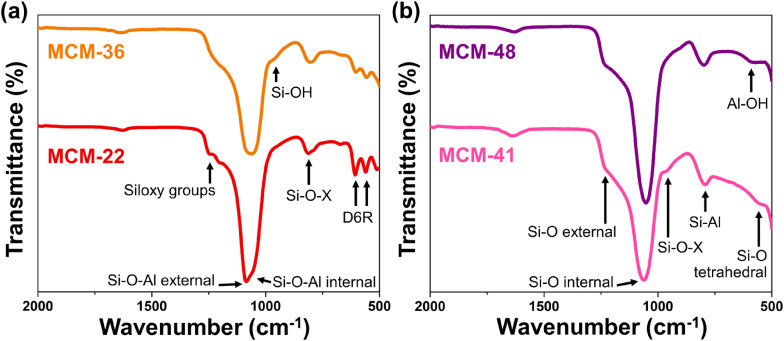
Functional groups of synthesized support materials (a) MWW-type zeolites and (b) mesoporous materials MCM-41 and MCM-48.

The IR spectra in Fig. 2a show that the internal and external asymmetric vibrations of Si–O–Al in the MCM-22 structure appear at 1084 and 1098 cm^−1^, respectively. The peak at 813 cm^−1^ corresponds to the symmetric stretching of Si–O–X (where X is Si or Al). Notably, the characteristic vibrations of the secondary building unit consisting of double six-membered rings (D6R) are observed at 567 and 614 cm^−1^, confirming the formation of the MWW topology.^20^ The IR spectrum of MCM-36 is generally similar to that of MCM-22, with a few key distinctions. The appearance of a peak at 962 cm^−1^ indicates the presence of Si–OH bonds^21^ attributed to silanol group formation within the pillared bridge. In addition, the sharper peak of the siloxy group at 1210 cm^−1^ in MCM-22 (ref. 22) becomes broader in MCM-36, suggesting a weakening of this bond. Finally, the reduced intensity of the D6R-related peaks indicates either an expansion or partial disruption of the zeolite framework, consistent with the X-ray diffraction results.

The vibration spectra of the hexagonally structured MCM-41 are presented in Fig. 2b. A peak observed at 1066 cm^−1^ and a broad shoulder at 1227 cm^−1^ correspond to the internal and external asymmetric stretching of Si–O, respectively,^23^ while the Si–O–X bonding is represented by a weak band at 970 cm^−1^.^24^ Notably, the peak at 799 cm^−1^ indicates the incorporation of alumina into the MCM-41 framework, as this peak appears at a lower wavenumber compared to that of pure silica MCM-41, which typically shows above 800 cm^−1^.^24^ Confirmation of the MCM-41 structure is further supported by the tetrahedral bending vibration of Si–O, observed at 537 cm^−1^.^23,25^ The IR spectrum of MCM-48 exhibits a similar pattern to that of MCM-41, although with some notable differences. The Si–O–X band is broader, reflecting lower structural regularity,^26^ which aligns with the cubic symmetry of MCM-48 in contrast to the hexagonal structure of MCM-41. The Si–Al peak also appears broader, and importantly, a distinct peak at 614 cm^−1^ assigned to Al–OH stretching^27^ confirms the presence of residual alumina in hydroxylated form, consistent with the XRD results indicating incomplete incorporation of alumina into the material framework.

### Porosity of microporous zeolites and mesoporous materials

**Fig. 3:**
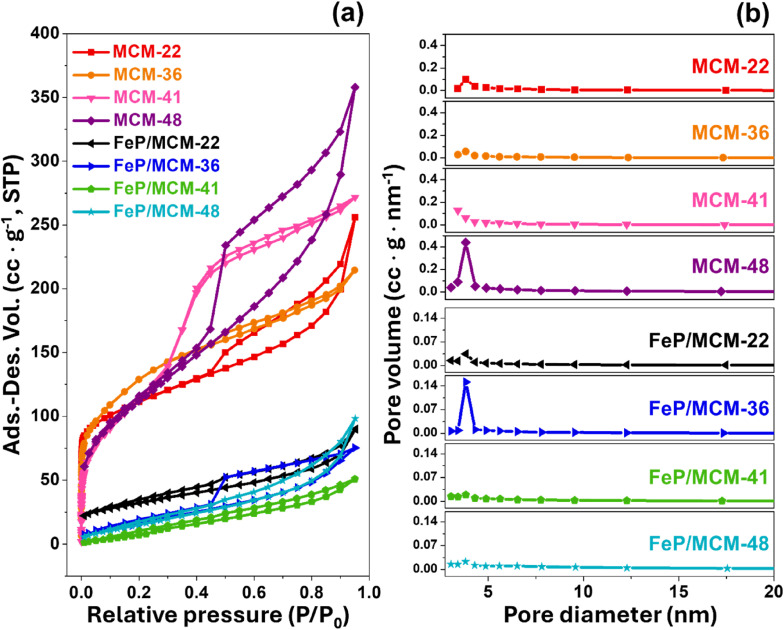
Surface and pore properties of synthesized materials and FeP catalyst support (a) N_2_-adsorption desorption isotherm and (b) Barrett–Joyner–Halenda (BJH) pore size distribution.

The N_2_ adsorption–desorption isotherms of all materials exhibited type IV hysteresis loop characteristics, as shown in Fig. 3a. Among the MWW-type zeolites, MCM-22 displayed an H4-type hysteresis loop,^28^ indicating the coexistence of micropores and mesopores in comparable proportions. The total adsorption–desorption volume of MCM-22 was higher than that of MCM-36, which can be attributed to its greater micropore content. This trend correlates well with the micropore volume data summarized in Table 1 and is consistent with previous findings.^9^ However, MCM-36 has a narrower hysteresis loop due to a more uniform distribution of mesopores, as confirmed by the smooth pore size distribution curve in Fig. 3b, indicating minimal variation in mesopore sizes.

**Table 1:** Surface areas and pore properties of microporous zeolite, mesoporous materials catalyst support

Samples	Surface area (m^2^ g^−1^)	Micropore volume (cc g^−1^)	Micropore area (m^2^ g^−1^)	Mesopore area (m^2^ g^−1^)	Average pore diameter (nm)
MCM-22	399	0.079	187	212	3.96
MCM-36	461	0.068	164	297	2.88
MCM-41	440	0	0	440	3.82
MCM-48	408	0	0	408	5.42

FeP/MCM-22	114	0.008	18	96	3.408
FeP/MCM-36	78	0	0	78	3.407
FeP/MCM-41	77	0	0	77	3.411
FeP/MCM-48	72	0	0	72	3.817

For mesoporous materials such as MCM-41 and MCM-48, the total adsorption–desorption volume was found to be higher than that of all MWW-type zeolites. MCM-41 exhibited a very narrow hysteresis loop, indicating the uniformity of its mesoporous structure. Meanwhile, MCM-48 showed the highest adsorption–desorption volume and the widest hysteresis loop. Despite having a smaller surface area than MCM-41, the average pore diameter of MCM-48 was significantly larger, as shown in Table 1. Consistent with the XRD results in Fig. 1b, the broad desorption hysteresis loop observed in the MCM-48 sample can be attributed to the presence of unconsumed Al(OH)_3_, or extra-framework alumina. This excess alumina may disrupt the meso-structure order, introduce defects in material, or cause partial pore blocking, leading to increased pore heterogeneity and delayed capillary condensation, which broadens the hysteresis loop.^29,30^ Additionally, as presented by Fig. 3b, MCM-48 has a much larger number of 3.8 nm-sized pores than other pore sizes, also more obvious than in other samples. This indicates that while the dominant mesopore size at 3.8 nm remains, other pore sizes may be blocked, contributing to the broadened hysteresis loop. This supports the presence of structural heterogeneity caused by residual alumina.

### Thermal stability of microporous zeolites and mesoporous materials

**Fig. 4:**
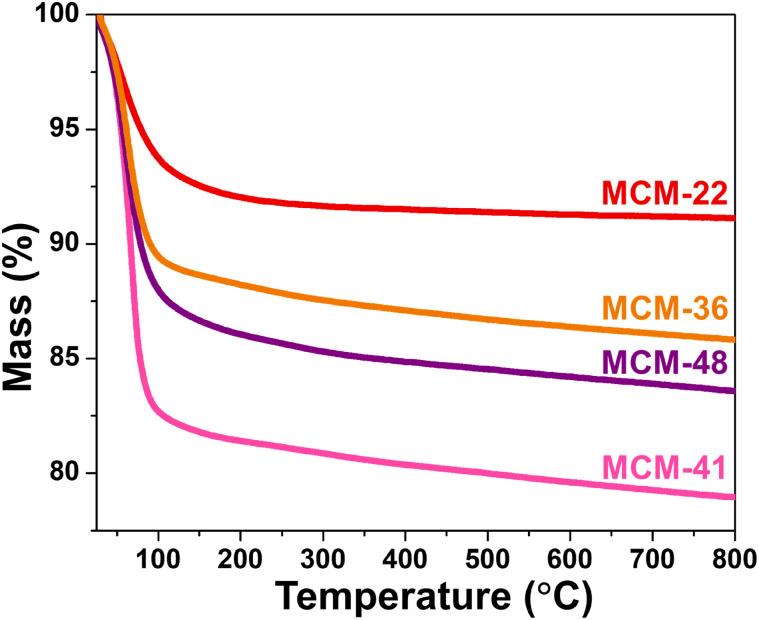
Thermal stability of synthesized support materials.

The TGA curves of all materials are shown in Fig. 4. All samples exhibited the highest mass reduction between approximately 30–150 °C, which is attributed to the loss of physically adsorbed humidity within the material's cavities. MCM-22 displayed the lowest mass reduction, around 8%, followed by MCM-36 (11%), MCM-48 (14%), and MCM-41 (18%). The mass reduction trend observed across the materials correlates with the mesopore area, suggesting that mesopores contribute more significantly to surface contact, while micropores have a less noticeable effect on this activity. Furthermore, MCM-22 demonstrated superior thermal stability, as indicated by the flatter curve with increasing temperature. This result is consistent with the XRD and FT-IR findings, which show the highest crystallinity and the strongest siloxy bonds in MCM-22, both of which play a crucial role in enhancing its thermal stability.^31^

### Acidity of microporous zeolites and mesoporous materials

**Fig. 5:**
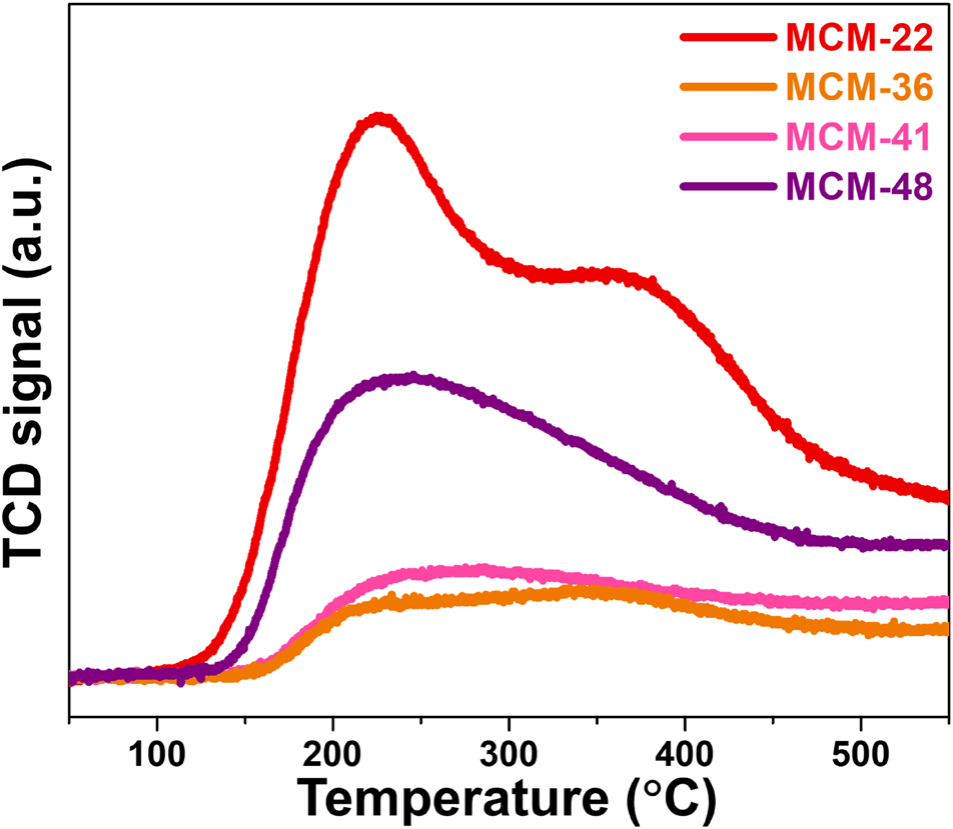
NH_3_-TPD profile of synthesized support materials.

The acidity of the materials was evaluated using NH_3_-TPD technique, with their profiles shown in Fig. 5. MCM-22 exhibited significant acidity, with both weak acid sites (0.42 mmol NH_3_ g^−1^) and moderate acid sites (0.336 mmol NH_3_ g^−1^), with the desorption peaks for these sites centered at approximately 220 °C and 330 °C, respectively.^9^ The desorption curve of MCM-36 also showed the presence of both weak and moderate acid sites, similar to MCM-22, but with a noticeable difference in the desorption amounts. The acidity of MCM-36 could be classified as weak acid sites (0.127 mmol NH_3_ g^−1^) and moderate acid sites (0.236 mmol NH_3_ g^−1^). The decrease in acidity observed in the MWW-type zeolites is consistent with the reduction in microporosity within the zeolite structure.^15^ The desorption curve for MCM-41 revealed a peak predominantly for weak acid sites,^32^ which aligns with the fact that it is a pure mesoporous material. The acidity content in MCM-41 (0.354 mmol NH_3_ g^−1^) is similar to that of MCM-36. In the case of MCM-48, the desorption profile is similar to MCM-41, showing only weak acid sites^33^ but with a significantly higher acidity content, which correlates with its larger average pore diameter, enhancing the accessibility of the acidic sites. However, the acidic sites in MCM-48 may originate in part from extra-framework alumina species such as Al(OH)_3_ (as observed by XRD and FT-IR results in Fig. 1 and 2), which are typically associated with Lewis acidity.^34^

Notably, compared with NH_3_-TPD data reported by Das *et al.*,^35^ the desorption peaks observed in our study are shifted to significantly higher temperatures. The above research reported NH_3_ desorption for weak acid sites below 435 K (∼162 °C) while the medium acid sites were centered at 537 K (∼264 °C) and the strong acid sites between 737–835 K (464–562 °C) for mesoporous zirconium oxophosphates. Similarly, the Brønsted-type acid sites in sulfonated porous polymers were classified a weak acid sites in the range of 30–150 °C, and the range of 182–250 °C was identified as moderate acid sites by Kundu and Bhaumik.^36^ In contrast, the synthesized materials in our study, particularly MCM-22 and MCM-36 zeolites, show a higher temperature of the NH_3_ desorption region (220 °C and 330 °C). This upward shift in desorption temperature may reflect the presence of stronger acid sites in our materials, likely associated with the Brønsted acidity arising from bridging hydroxyl groups (Si–OH–Al) within the framework.^37^ The tighter proton binding in these sites results in stronger NH_3_ adsorption.

### Morphology of microporous zeolites and mesoporous materials

**Fig. 6:**
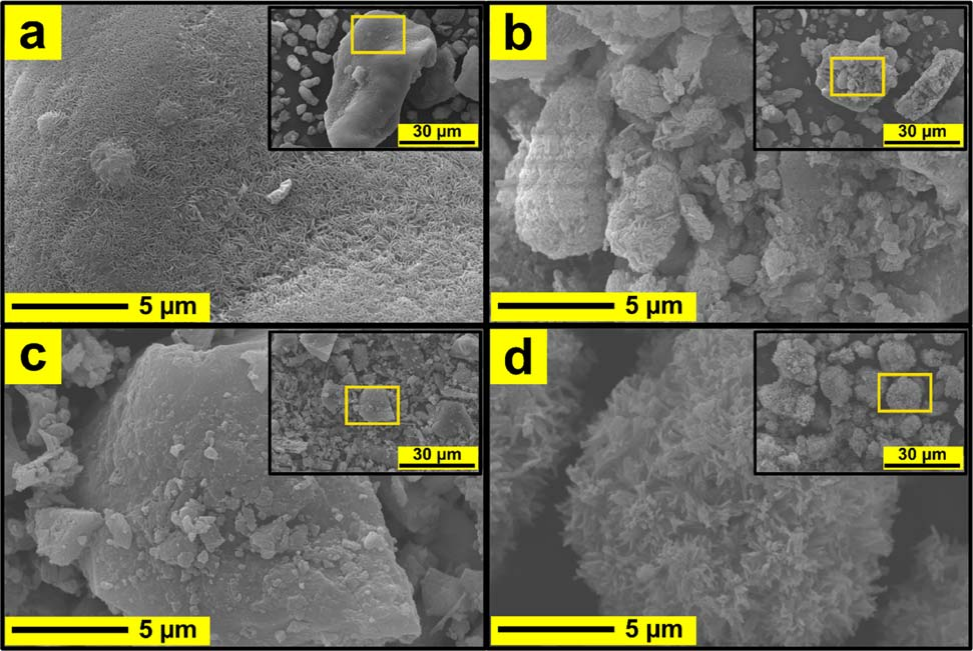
SEM image of synthesized support materials (a) MCM-22, (b) MCM-36 (c) MCM-41, and (d) MCM-48. The large image is a 200 00× magnification image while the small image is a 5000× magnification.

The morphology of the synthesized materials is illustrated in Fig. 6. MCM-22 exhibits a broad surface with uniformly distributed, orderly small petal-like structures.^9^ At 5000× magnification, MCM-36 appears as a coarser aggregate than MCM-22, while at 200 00×, it reveals a sheet-like morphology rather than a continuous field, with individual sheet thicknesses of less than 100 nm.^15^ MCM-41 is composed of small, relatively spherical particles that are tightly packed at 20 000× magnification. At lower magnification (5000×), these particles tend to aggregate into larger clusters, consistent with many previous reports.^38,39^ However, it is noteworthy that the small spherical particles in this study appear more abundant and better dispersed. MCM-48 shows well-formed, somewhat rounded particles with a fluffy texture.^40^ At higher magnification, its surface reveals numerous small, frost-like crystal clusters scattered throughout.

### Catalytic performance of FeP support catalysts

At the outset, the composition of palm olein oil was investigated by transforming triglyceride to fatty acid methyl esters (FAMEs) according to a method of Sukkathanyawat *et al.*^41^ The fatty acid composition of the oil was found to be as follows: lauric acid (C12:0) – 0.5%; myristic acid (C14:0) – 2.1%; palmitic acid (C16:0) – 33.0%; palmitoleic acid (C16:1) – 0.4%; margaric acid (C17:0) – 0.4%; stearic acid (C18:0) – 9.9%; oleic acid (C18:1) – 27.4%; linoleic acid (C182) – 24.1%; eicosanoic acid (C20:0) – 1.3%; and eicosenoic acid (C20:1) – 0.8%.

**Fig. 7:**
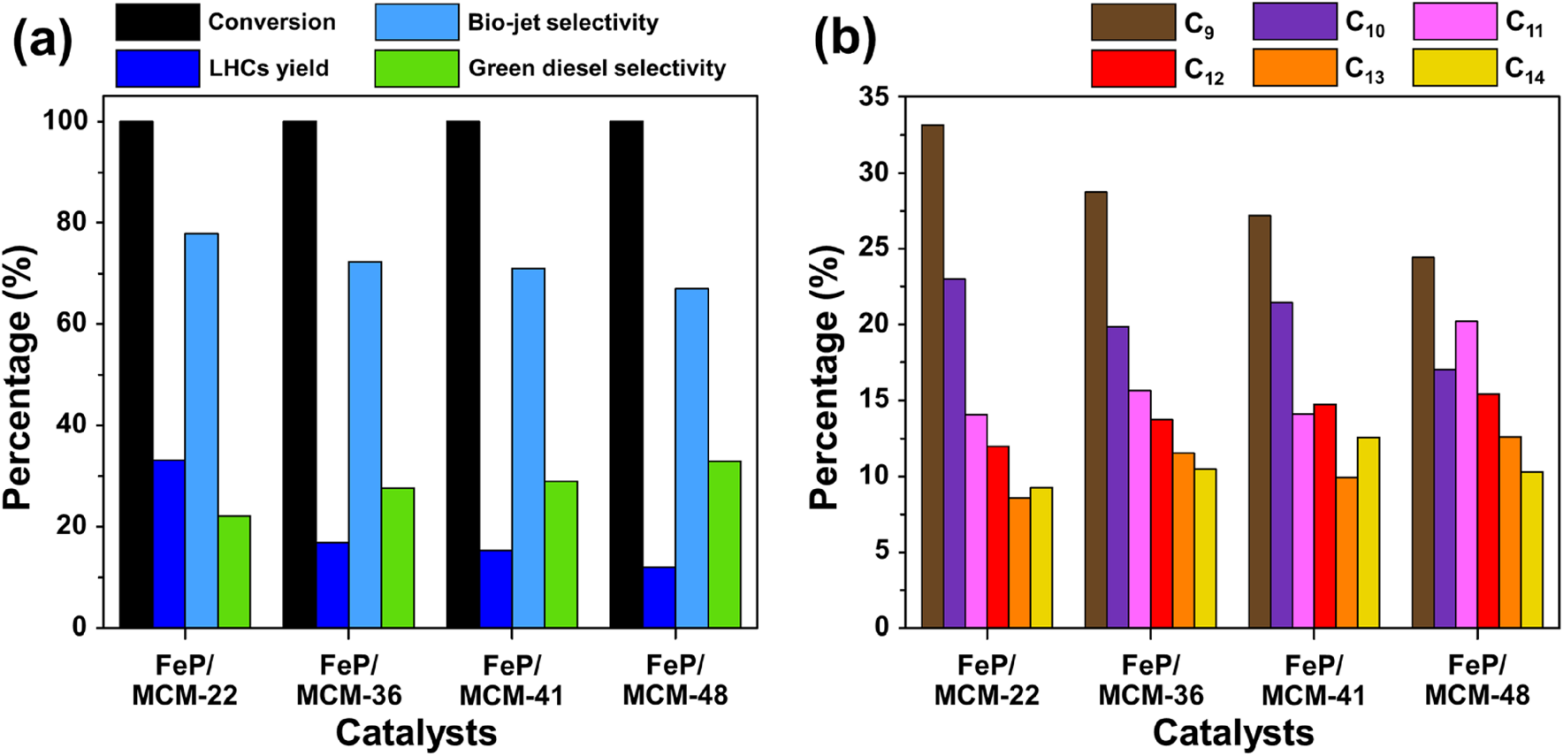
Catalytic ability of FeP supports catalyst (a) product portfolio and (b) *C*-number in bio-jet fuel formulation.

The performance of all catalysts in the hydrocracking of palm oil is presented in Fig. 7. As shown in Fig. 7a, all catalysts achieved 100% palm oil conversion. The highest yield of liquid hydrocarbons (LHCs) was obtained using the FeP/MCM-22 catalyst, reaching approximately 33%. A significant decrease in LHCs yield was observed when FeP/MCM-36 was used, with a yield of about 17%. Further reductions were found with FeP/MCM-41 and FeP/MCM-48, yielding approximately 15% and 12%, respectively, although the differences were less pronounced. The trend in bio-jet selectivity followed that of LHCs yield, with FeP/MCM-22 providing the highest selectivity at around 78%. In contrast, green diesel selectivity increased in the order of FeP/MCM-36, FeP/MCM-41, and FeP/MCM-48. According to Fig. 7b, FeP/MCM-22 also demonstrated outstanding performance in producing C_9_ and C_10_ hydrocarbons, accounting for approximately 33% and 23%, respectively. These results are attributed partly to the excellent intrinsic properties of iron phosphide, which favor the formation of light hydrocarbon molecules,^4^ and partly to the strong acidity of the MCM-22 zeolite. The notable presence of medium-strength acid sites and an adequate number of acidic sites is known to promote hydrocracking activity.^42^ The high activity and selectivity observed for the FeP/MCM-22 catalyst can be further explained by the abundance of Brønsted acid sites, associated with bridging hydroxyl groups (Si–OH–Al) within the MWW-type framework.^37^ These Brønsted acid sites are well recognized for their ability to facilitate C–C bond cleavage during hydrocracking,^43^ enhance hydrocracking activity.^44^ The NH_3_-TPD results (in Fig. 5) confirm the existence of medium-strength Brønsted acid sites in MCM-22, consistent with its superior cracking performance and product selectivity. The synergistic effect between the selective hydrogenolysis capability of FeP and the moderate Brønsted acidity of MCM-22 promotes both efficient cracking and selective production of bio-jet range hydrocarbons. FeP/MCM-36 shows a lower ability according to the similar trend of acid amount. The lower performance of FeP/mesoporous supports tends to decrease according to acid strength. Particularly, in the case of FeP/MCM-48, the presence of extra-framework alumina species can result in the presence of Lewis acid sites in this support. These Lewis sites are generally less effective in hydrocracking and are more prone to coke formation, thus leading to reduced LHCs yields and selectivity.^45^

### Reduction behavior of FeP catalyst on different supports

**Fig. 8:**
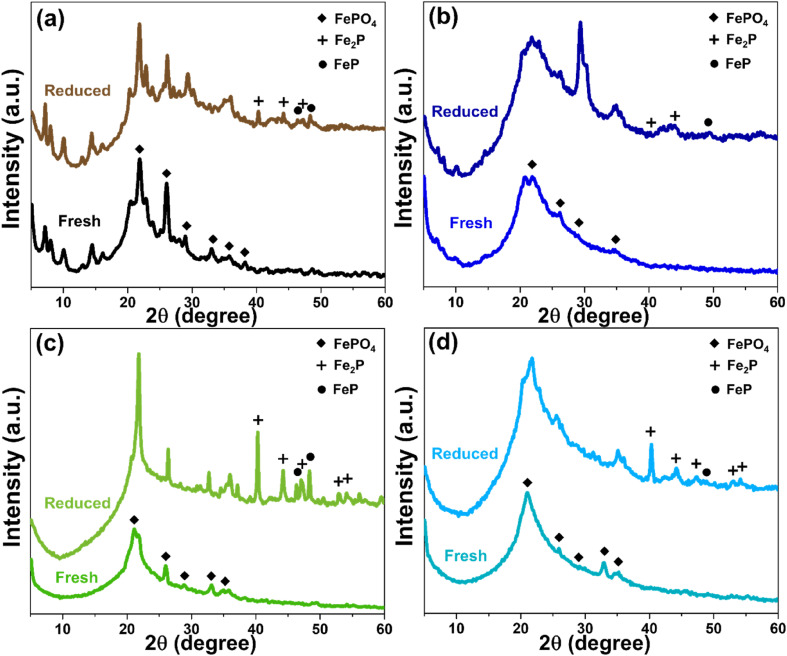
XRD pattern of FeP support catalysts comparing fresh and reduced (a) FeP/MCM-22, (b) FeP/MCM-36, (c) FeP/MCM-41, and (d) FeP/MCM-48.

The structure, porosity, and acidity of each support materials also significantly influenced the reduction behavior of FeP deposited on their surfaces. The X-ray diffractogram patterns of all four catalysts, in both fresh and reduced forms, are presented in Fig. 8. Initially, iron phosphate existed predominantly as FePO_4_, and after the reduction treatment, iron phosphide was formed mainly in the phases of FeP and Fe_2_P.^4,46^ However, in the case of MWW-type zeolites, particularly MCM-22 and MCM-36, the FePO_4_ diffraction peaks were partially overlapped and obscured by the intense peaks of the crystalline zeolite framework. As shown in Fig. 8a, although both FeP and Fe_2_P phases were detected after reduction, a portion of the FePO_4_ phase in MCM-22 zeolite remained unconverted. Considering the % pore consumption, it was found that most of the FePO_4_ was loaded in the micropores, as summarized in Table 1. In the case of FeP/MCM-36, as shown in Fig. 8b, the XRD peak intensity changed significantly compared to the pure MCM-36 zeolite (Fig. 1), indicating a high loading of FePO_4_ within the zeolite structure. This observation is consistent with the surface area and pore volume analysis in Table 1. The data further reveal that the micropores of MCM-36 were completely filled with FePO_4_, and mesopore consumption exceeded 70%, unlike in MCM-22. This difference can be attributed to the higher acidity of MCM-22, which limits FePO_4_ synthesized from phosphoric acid, and the significantly greater mesopore volume of MCM-36, which allows higher FePO_4_ uptake. After reduction, FeP and Fe_2_P phases appeared in lower intensity for FeP/MCM-36 compared to FeP/MCM-22, while the FePO_4_ peak remained clearly dominant. These results suggest that FeP/MCM-36 undergoes less extensive H_2_ reduction during heat treatment, leading to the preservation of highly crystalline FePO_4_ in the structure. The blockage in the micropores of MCM-36 is supported by the N_2_ adsorption–desorption isotherm in Fig. 3a, which shows a significantly widened hysteresis loop when FePO_4_ is loaded. This observation is consistent with the BJH pore size distribution (Fig. 3b), indicating that FePO_4_ likely occupied other mesopore sizes, possibly larger or smaller than ∼3.8 nm, contributing to increased pore heterogeneity and a wider hysteresis loop. Notably, the average pore size of FePO_4_/MCM-36 increased after loading, in contrast to the other supports, which exhibited a reduction in average pore size upon FePO_4_ incorporation.

MCM-41 is a purely mesoporous material. Owing to its low acidity, high surface area, and large pore size, it allows a high FePO_4_ loading, with pore consumption exceeding 80%. The broad and comprehensive pore-filling range of FePO_4_, as shown in Fig. 3, facilitates effective reduction. As observed in Fig. 8c, substantial amounts of FeP and Fe_2_P phases are formed; however, FePO_4_ also remains, exhibiting an even more pronounced peak after heat treatment. This residual FePO_4_ likely results from the high loading within the mesopores, which promotes clustering. For FeP/MCM-48, after reduction, the catalyst predominantly exhibits the Fe_2_P phase, with only a minimal amount of FeP remaining (Fig. 8d), in contrast to FeP/MCM-41. This behavior may be attributed to the significantly large average pore size of MCM-48, which facilitates improved surface-level reduction. However, the complex pore architecture and defects of the MCM-48 structure can contribute to a lower overall reduction efficiency. Additionally, the large average pore size may promote extensive FePO_4_ clustering, which also influences the reduction efficiency. In addition, the distribution characteristics of FeP particles on each support material were examined by TEM at both 150 000× and 50 000× magnifications, as shown in Fig. S1.† Consistent with the surface area and porosity results, FeP particles supported on MCM-22 were well and uniformly dispersed. At 150 000× magnification (Fig. S1a†), individual FeP particles were barely visible, but this suggests excellent dispersion of metal particles throughout the MCM-22 framework.^47^ This good dispersion was further confirmed at 50 000× magnification (Fig. S1b†), where no localized clusters were observed. In the case of FeP/MCM-36, individual FeP particles approximately 10 nm in size were observed (lighter spots), while clustered regions (darker spots) were about 14 nm in diameter at high magnification (Fig. S1c†). More extensive agglomeration was clearly visible at lower magnification (Fig. S1d†). Similarly, FeP particles on MCM-41 (Fig. S1e†) showed comparable particle sizes to the previous sample and exhibited significant clustering, which was also confirmed at lower magnification (Fig. S1f†). Remarkably, FeP particles supported on MCM-48 appeared to be significantly smaller, typically below 10 nm. However, dark areas indicating patchy agglomeration were clearly apparent at both magnifications (Fig. S1g and h†).

**Fig. 9:**
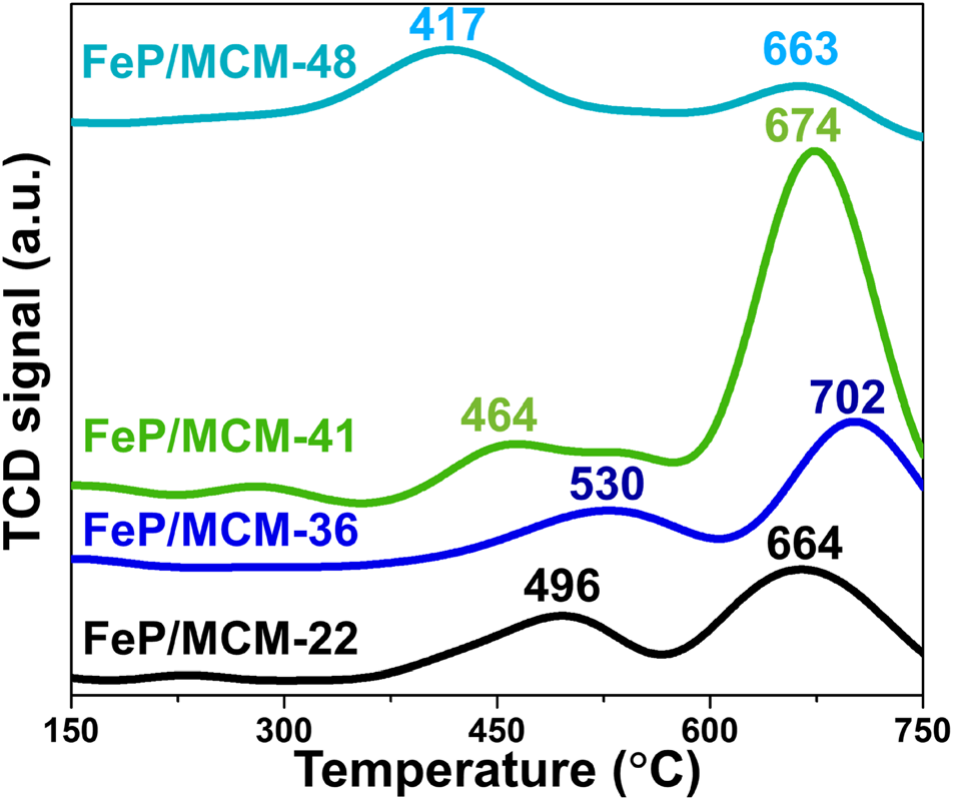
H_2_-TPR profile of FeP catalysts support.

To elucidate the above phenomenon in greater detail, the reduction behavior of all FeP/support catalysts can be explained through the H_2_-TPR profiles shown in Fig. 9. All samples exhibit two main reduction peaks: the first peak at lower temperatures (approximately 400–550 °C), attributed to the reduction of Fe^3+^ from FePO_4_, and the second peak at higher temperatures (approximately 660–700 °C), corresponding to the reduction of FeP to Fe_2_P (Fe^2+^).^48,49^ The reduction of iron phosphate on MWW-type zeolites begins at relatively higher temperatures, which is consistent with the presence of micropores in this zeolite family and the incorporation of FePO_4_ into these micropores. When comparing FeP/MCM-22 and FeP/MCM-36, the reduction peak of FePO_4_ in FeP/MCM-36 appears broader and shifts to higher temperatures. This behavior aligns with the clogging of micropores by iron phosphate and the higher total FeP loading, both of which contribute to a delayed reduction of FeP to Fe_2_P.

The reduction of FePO_4_ in FeP/MCM-41 occurs at 464 °C, which can be attributed to the large pore size and high surface area of this mesoporous material, facilitating effective surface dispersion of iron phosphate. Additionally, the incorporation of FePO_4_ into the wide and uniform mesopores (see Fig. 3) promotes significant reduction of FeP to Fe_2_P, which is consistent with the crystallographic results shown in Fig. 8. MCM-48 exhibits the poorest reduction behavior, although the initial reduction step occurs at the lowest temperature, likely due to its significantly large pore size. However, the subsequent reduction step occurs only to a limited extent, even though it appears at a lower temperature compared to the other samples. Interestingly, this observation contrasts with the X-ray diffractogram after reduction (Fig. 8), which reveals substantial crystallization of Fe_2_P. This discrepancy suggests that the complex three-dimensional interconnected pore network of MCM-48 may impede the release of H_2_ due to its strong H_2_ confinement capability,^50^ especially in the presence of metal dopants,^51,52^ thereby affecting the TCD signal response.

### Catalyst's reusability

**Fig. 10:**
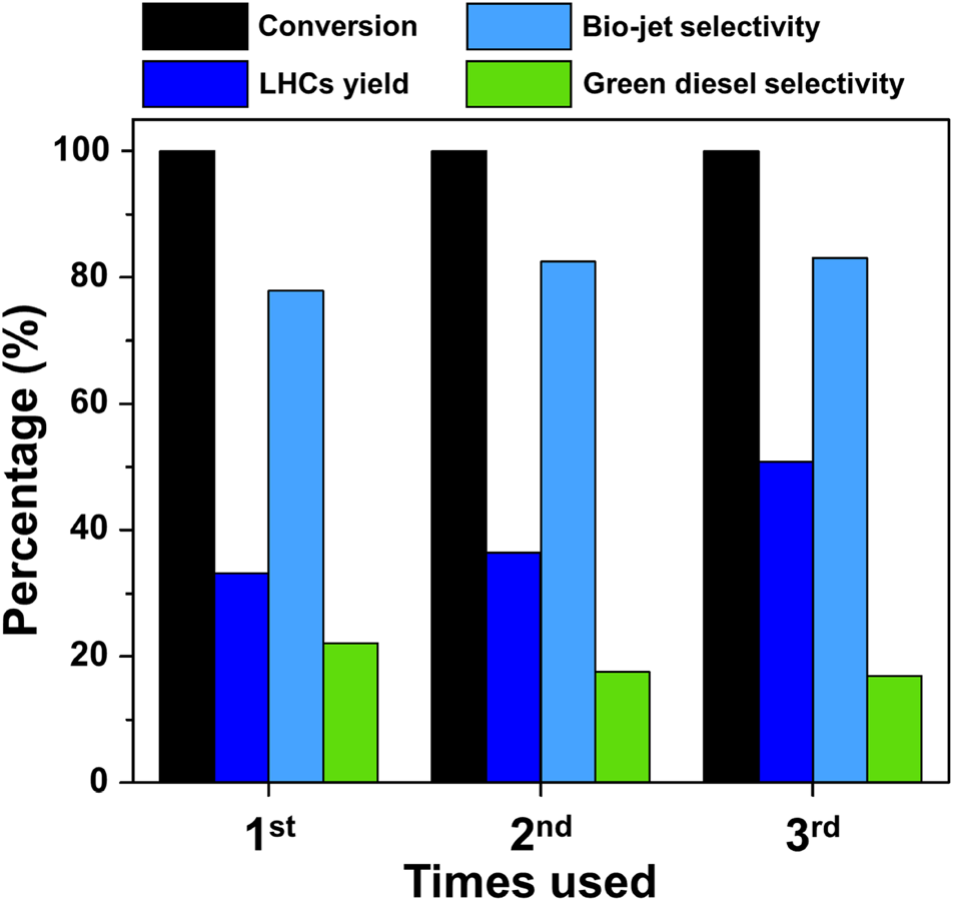
Reusability of FeP/MCM-22 catalyst.

**Fig. 11:**
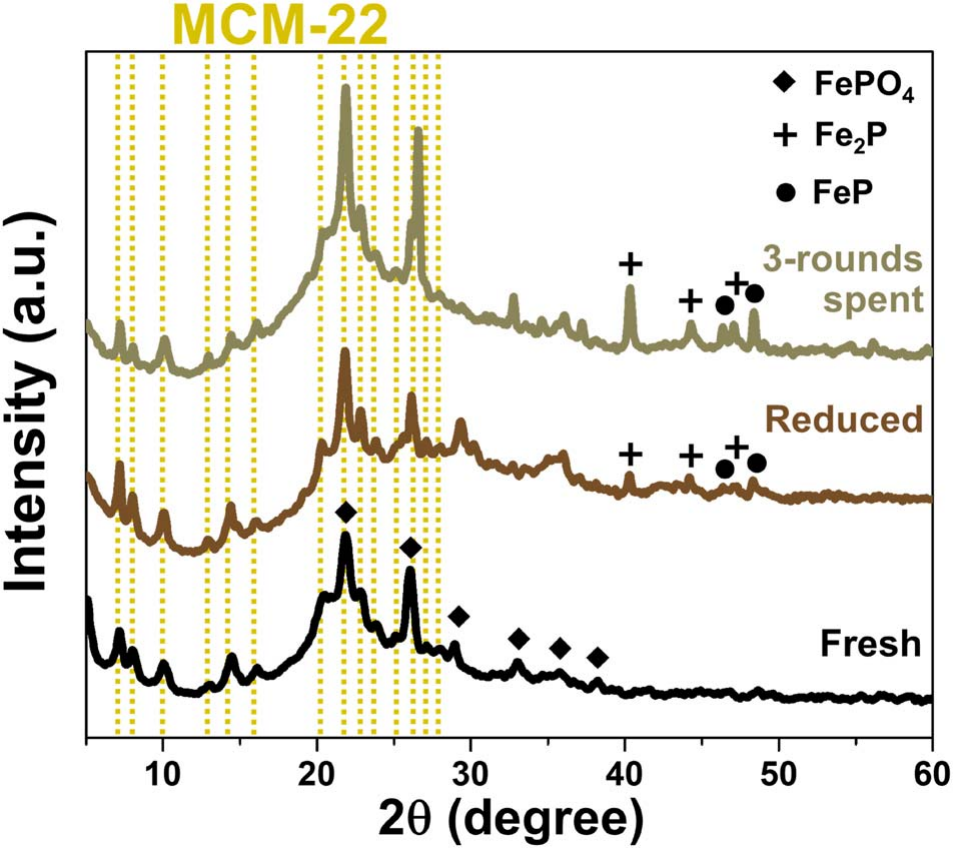
XRD pattern of FeP/MCM-22 catalysts in forms of fresh, reduced, and 3-rounds spent.

The stability of the FeP/MCM-22 catalyst is presented in Fig. 10. This catalyst exhibited a relatively low yield of liquid hydrocarbons (LHCs) and low bio-jet selectivity during its first use. Nevertheless, the recycling of this catalyst reveals an interesting trend. In the second cycle, both LHCs yield and bio-jet selectivity were enhanced, reaching approximately 36% and 82%, respectively. In the third cycle, the LHCs yield increased further to about 50%, while the bio-jet selectivity remained nearly unchanged. The effect of catalyst reuse in the hydrocracking process can be further explained by Fig. 11, which shows the X-ray diffractograms of FeP/MCM-22 in fresh, reduced, and three-times-spent forms. The spent catalyst exhibits significantly more intense peaks of both FeP and Fe_2_P phases, indicating enhanced metal crystallization and transformation during the repeated reaction cycles. In conjunction with the EDS results shown in Fig. S2,† which indicate a substantial decrease in oxygen and nearly a twofold increase in the surface content of Fe and P atoms after three cycles of catalyst reuse, these findings suggest that repeated reduction cycles promote further oxygen removal and the formation of more FeP and Fe_2_P phases. The reduction step prior to each reaction cycle enhances the formation of these phosphide phases, and once generated, the iron phosphide phases are difficult to reverse-oxidize to the phosphate form due to the inherent ability of phosphorus to inhibit Fe ion oxidation.^53,54^ This highlights the stabilizing role of phosphorus in protecting the Fe phase. The increased amount of Fe species on the surface correlates with improved bio-jet selectivity; however, after multiple reductions and cycles of reuse, this selectivity tends to plateau, likely due to phase stabilization and coke formation.^55^ Finally, the enhanced reduction of FePO_4_ and the increased dispersion of Fe and P species on the catalyst surface may help alleviate micropore blockage, thereby exposing more acidic sites and contributing to the observed increase in LHCs yield.

## Conclusion

Among the four support materials synthesized in this study, comprising two microporous zeolites and two mesoporous materials, MCM-22 zeolite exhibited the highest surface area of micropores (approximately 187 m^2^ g^−1^) and the greatest thermal stability, attributed to its high crystallinity and ability to form strong functional groups. Furthermore, it possessed significantly higher acidity than the other three supports (MCM-36, MCM-41, and MCM-48). These characteristics strongly influenced the performance of FeP-based catalysts in the hydrocracking of palm oil to bio-jet fuel. The microporous MWW-type zeolites outperformed the mesoporous supports, with FeP/MCM-22 demonstrating the highest catalytic efficiency, yielding 33% liquid hydrocarbons and achieving 78% bio-jet range selectivity. Reusability testing showed that after three cycles of reuse, both catalytic performance parameters (LHCs yield and bio-jet selectivity) improved to approximately 50% and 83%, respectively. These enhancements were attributed to increased accessibility of acidic sites within the zeolite framework and a greater formation of active iron phosphide phases. The excellent acidity and microporosity of MCM-22 not only prevent excessive loading of iron phosphide but also actively contribute to the catalytic process, particularly in improving liquid hydrocarbon yields.

## Author contributions

Worapak Tanwongwan: conceived and designed the experiments; performed the experiments; analyzed and interpreted the data; wrote the paper. Ruttasart Sartsamai: performed the experiments. Nuwong Chollacoop: Kajornsak Faungnawakij: analyzed and interpreted the data. Rungnapa Kaewmeesri: Suttichai Assabumrungrat: Masayoshi Fuji: analyzed and interpreted the data; contributed reagents, materials, analysis tools or data. Apiluck Eiad-ua: conceived and designed the experiments; contributed reagents, materials, analysis tools or data; wrote the paper.

## Conflicts of interest

There are no conflicts to declare.

## Supplementary Material

RA-015-D5RA02133B-s001

## Data Availability

Data included in article/ESI†/referenced in article.
