# Supplementary material for: Potential of advanced microporous zeolites and mesoporous materials derived from natural precursors as supports for iron phosphide catalysts in bio-jet fuel production from palm oil (Elaeis guineensis)
Source: RSC Adv. 2025 Jun 10;15(25):19714–25. doi: 10.1039/d5ra02133b (PMC12151145; doi:10.1039/d5ra02133b)
Supplement: RA-015-D5RA02133B-s001 [file RA-015-D5RA02133B-s001.pdf]

Supplementary Information (SI) for RSC Advances.
This journal is © The Royal Society of Chemistry 2025

Revised Supplementary Information

Potential of advanced microporous zeolites and mesoporous materials derived from natural precursors as support for iron phosphide catalysts in bio-jet fuel production from palm oil *(Elaeis guineensis)*

*Worapak Tanwongwan[a], Ruttasart Sartsamai[a], Rungnapa Kaewmeesri[b], Kajornsak Faungnawakij[b], Nuwong Chollacoop[c], Suttichai Assabumrungrat[d], Masayoshi Fuji[e], and Apiluck Eiad-ua[a]**

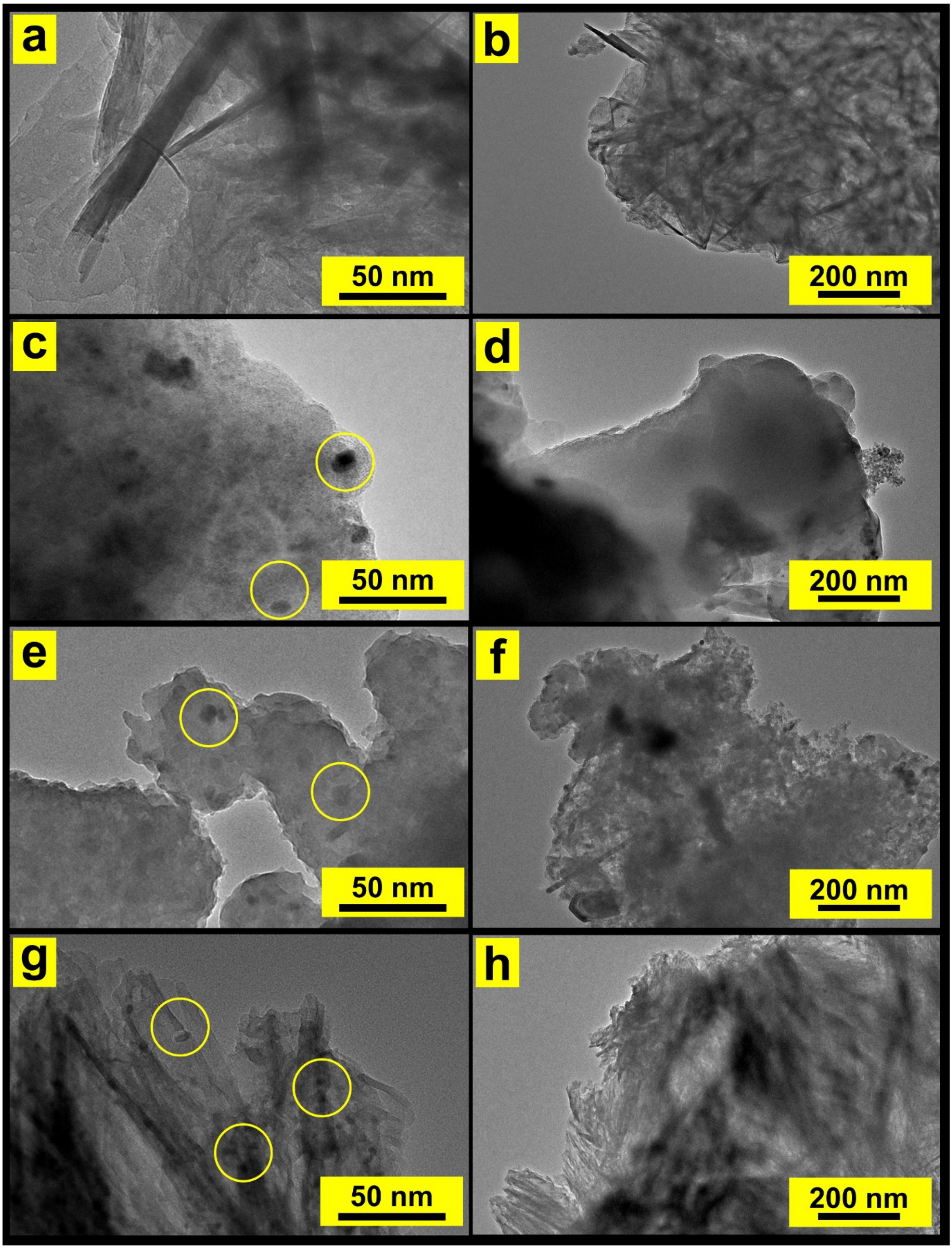

Figure S1 | Transmission electron micrograph of all catalyst. TEM images at 150,000×, and 50,000× magnifications of reduced FeP catalysts dispersed on different zeolites include (a-b) FeP/MCM-22, (c-d) FeP/MCM-36, (e-f) FeP/MCM-41, and (g-h) FeP/MCM-48.

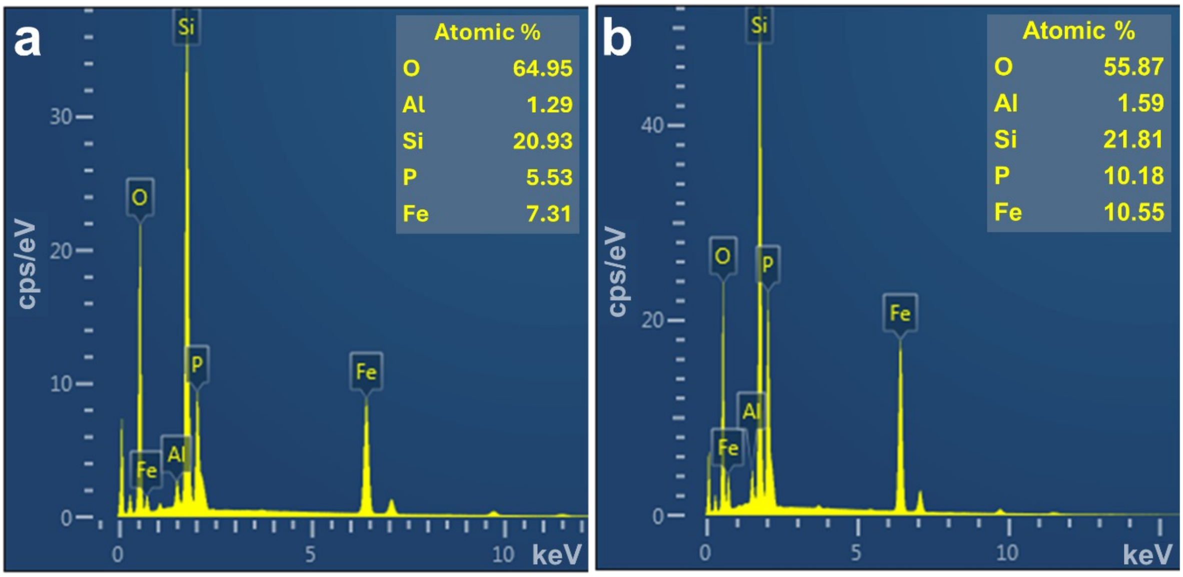

Figure S2 | Energy Dispersive X-ray spectrum. EDS graph of FeP/MCM-22 catalyst includes (a) fresh and, (b) after 3 catalytic cycles.
